# Separation and Recycling of Concentrated Heavy Metal Wastewater by Tube Membrane Distillation Integrated with Crystallization

**DOI:** 10.3390/membranes10010019

**Published:** 2020-01-20

**Authors:** Xiang-Yang Lou, Zheng Xu, An-Ping Bai, Montserrat Resina-Gallego, Zhong-Guang Ji

**Affiliations:** 1 National Engineering Lab. of Biohydrometallurgy, GRINM Technology Group Co., Ltd., Beijing 101407, China; Xiangyang.Lou@uab.cat (X.-Y.L.); xzh63@126.com (Z.X.); 2 GTS Research Group, Department of Chemistry, Faculty of Science, Universitat Autònoma de Barcelona, 08290 Bellaterra, Spain; montserrat.resina@uab.cat; 3 General Research Institute for Nonferrous Metals, Beijing 100088, China; 4 GRINM Resources and Environmental Tech. Co., Ltd., Beijing 101407, China; 5 Beijing Vocational College of Labor and Social Security, Beijing 102200, China; baianping1992@163.com

**Keywords:** wastewater treatment, membrane distillation, crystallization, heavy metals, concentrated solutions, membrane fouling

## Abstract

Tube membrane distillation (MD) integrated with a crystallization method is used in this study for the concurrent productions of pure water and salt crystals from concentrated single and mixed system solutions. The effects of concentrated Zn^2+^ and Ni^2+^ on performance in terms of membrane flux, permeate conductivity, crystal recovery rates, and crystal grades are investigated. Preferred crystallization and co-crystallization determinations were performed for mixed solutions. The results revealed that membrane fluxes remained at 2.61 kg·m^−2^·h^−1^ and showed a sharp decline until the saturation increased to 1.38. Water yield conductivity was below 10 μs·cm^−1^. High concentrated zinc and nickel did not have a particular effect on the rejection of the membrane process. For the mixed solutions, membrane flux showed a sharp decrease due to the high saturation, while the conductivity of permeate remained below 10 μs·cm^−1^ during the whole process. Co-crystallization has been proven to be a better method due to the existence of the SO_4_^2−^ common-ion effect. Membrane fouling studies have suggested that the membrane has excellent resistance to fouling from highly concentrated solutions. The MD integrated with crystallization proves to be a promising technology for treating highly concentrated heavy metal solutions.

## 1. Introduction

The increase of heavy metal wastewater has not only strained already limited freshwater but also caused the waste of heavy metal resources all over the world [1]. At the same time, electroplating, electrolysis, and other industrial processes are generating highly concentrated zinc and nickel wastewater every year [2]. These solutions are characterized by their high acidity, strong corrosion capacity, and elevated metal concentrations. Nowadays, the industry uses precipitation as a low-cost and straightforward method to treat this wastewater, but unfortunately, it produces a large amount of high-risk sludge in the meantime [3,4]. The traditional evaporation crystallization method takes lots of energy and needs reactors with excellent resistance to corrosion [5]. Besides, the most desirable goal of substantial metal wastewater treatment is to achieve the recovery of valuable metals and water. Therefore, it is essential to develop new technologies to deal with this kind of wastewater [6].

Over the past thirty years, membrane-based separation processes have emerged as the most promising techniques in the wastewater treatment field, owing to the significant evolution of membrane technologies [7,8,9]. Among membrane technologies, membrane distillation (MD) is a thermally driven desalination process in which a porous hydrophobic membrane is employed as a physical barrier between the hot feed, where the volatile compounds are evaporated, and the distillate side, where the diffused vapors are condensed [10]. The driving force of MD is the difference of vapor pressure on both sides of the membrane. Therefore, it is unnecessary to heat the feed solution to boiling temperature. In the MD process, solar, wind, and other clean energies or waste heat can be used [11,12]. Besides, due to its mild operating conditions and excellent sealing properties, MD is suitable for the treatment of toxic and harmful concentrated heavy metal or radioactive wastewater. On the other hand, crystallization is a very mature technology that has been widely used in the industrial sector [13]. A complete set of the theoretical and technical system is formed by the application of cooling crystallization to the recovery of solute in a saturated solution. Membrane distillation integrated with crystallization is a combination of these two technologies. Its principle is to remove the volatile solvent (generally water) by the MD process, concentrate the solution to reach saturation, then allow cooling crystallization to do the solid–liquid separation to get crystals [14,15]. 

The treatment of heavy metal solutions by MD has been reported. Yuan et al. [16] utilized the hollow fiber membrane in MD to deal with multiple heavy metal dilute solutions and found that copper, nickel, and zinc ions have no significant effects on the stability of flux and the quality of water yield. There are also some reports on the research of concentrated solutions by MD integrated with crystallization technology [17,18,19,20,21,22]. For instance, Edwie et al. [19] used three different hollow fiber membranes to treat saturated NaCl solution and found that a smaller membrane pore size and a more compact membrane surface are beneficial for the stability and continuity of the MD integrated with crystallization process. Chan et al. [22] utilized a flat sheet membrane to treat NaSO_4_ and NaCl single element solutions, respectively, and found that temperature and saturation levels are critical operating parameters in the MD integrated with crystallization process. On the other hand, the treatment of mixed heavy metal solutions by means of MD integrated with crystallization technology has rarely been reported. Besides, when treating concentrated solutions, a rapid flux decline is usually observed due to crystal deposition and scale formation on the membrane surface, which reduces membrane permeability. It is found that hollow fiber and flat membranes are generally used in the existing MD integrated with crystallization processes. The filament diameter of the hollow fiber membrane is small, so it is easy to crystallize at the fiber inlet when treating highly concentrated solutions. Moreover, the flow pattern of the flat membrane is difficult to control and it is easy to form crystallization at the membrane surface. All these limitations restrict the development of MD integrated with crystallization technology; in order to overcome them, tube membranes were used in this work.

This study, therefore, aims to investigate the efficiency of MD integrated with crystallization technology in treating concentrated heavy metal wastewater. Concentrated single zinc and nickel system solutions and mixed system solutions were used to get pure crystals and distilled water. As the foremost concern, membrane fouling was investigated by using SEM and EDS. 

## 2. Materials and Methods 

### 2.1. Materials

The reagents used in this study were ZnSO_4_·7H_2_O and NiSO_4_·6H_2_O. All are analytical grade and purchased from Tianjin Guangfu Fine Chemical Research Institute, Tianjin, China. As it is shown in Table 1 [23], these reagents have different solubilities at 10 °C (cooling crystallization temperature) and 65 °C (heat side temperature of MD). According to this condition, it is possible to cool crystallization to obtain solid salt before MD is blocked by membrane fouling. 

For the mixed system, if one reagent reaches its saturation point at 65 °C, while the other is not saturated at 10 °C, it would thus be possible to separate one from the other. Otherwise, co-crystallization will be a better option. Without considering the common-ion effect, three different ratios of ZnSO_4_ and NiSO_4_ were selected, and three different methods were chosen to treat the mixed solutions in this study accordingly, as listed in Table 2.

PTFE tube membranes were kindly supplied by Huzhou Sano Environmental Technology, China. The membrane module is a self-made air gap type component. 

### 2.2. Membrane Characterizations

The diameter and thickness were measured using a Vernier caliper. The measurement of the advancing contact angle was performed using a dynamic contact angle meter (DCAT21, Data Physics, Filderstadt, Germany). The membrane nominal and maximum pore sizes were conducted with a capillary flow parameter (Proulx 1000, Porometer NV, Nazareth, Belgium). The mechanical properties of the tube membrane were characterized in terms of strain and tensile stress at break using an electronic fabric strength tester (YG065CQS/pc, Changzhou No.1 Textile Equipment Co., Ltd., Changzhou, China). Table 3 shows the parameters of the membrane material and module. 

### 2.3. MD Integrated with Crystallization Experiments

**Figure 1:**
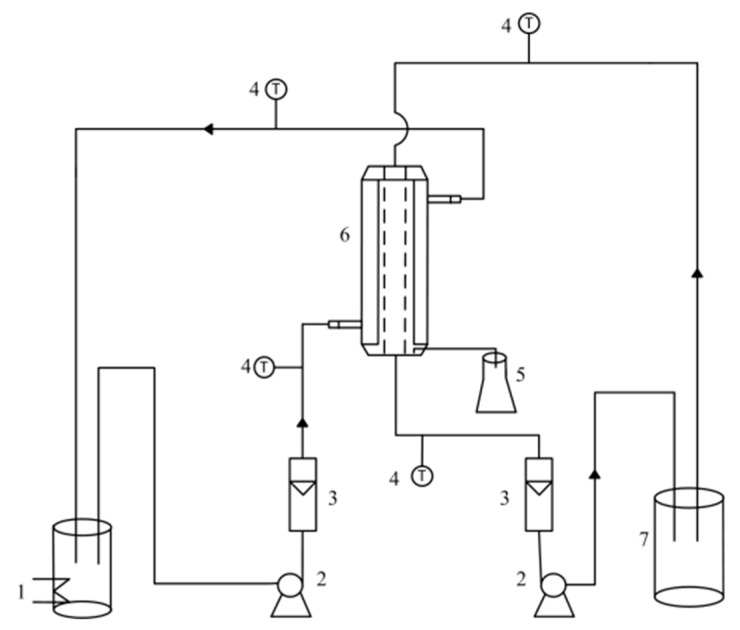
Experimental apparatus diagram of air gap membrane distillation system. (**1**) Thermostatic hot bath; (**2**) magnetic pump; (**3**) liquid flowmeter; (**4**) thermometer; (**5**) product water collector; (**6**) membrane module; (**7**) thermostatic cold bath.

MD integrated with crystallization experiments were carried out by a laboratory-scale setup. The experimental apparatus diagram of air gap membrane distillation is shown in Figure 1. The membrane modules were self-made, and there were tube glasses, tube membranes, and tube condensers from the outside to the inside. During the MD process, hot feed flows between the membrane and the glass, and cold water flows inside the condense tube. There was not a certain width of the gap in this case. A 1000 mL beaker flask was used as a cooling mold. 

Prepared feed solution is heated by a thermostatic hot bath, then flows through the membrane module driven by a magnetic force pump and recirculates on the module’s hot side. The same procedure is followed for the chilled water system. The vapor passing through the membrane is condensed and recovered on the cold side. As the experiment goes on, when the feed solution is supersaturated, the concentrated solution is pumped out to the mold and cooled to crystallize. Finally, crystallization products are obtained by solid-liquid separation. The remaining crystalline solution returns to the MD unit and continues to concentrate. 

According to previous experiments, the optimized average operating conditions are as follows: temperature of the hot side, 65 °C; temperature of the cold side, 20 °C; velocity of the hot side, 0.16 m·s^−1^; velocity of the cold side, 0.26 m·s^−1^; temperature of the cooling crystallization, 10 °C; time of cooling crystallization, 8 h.

### 2.4. MD Integrated with Crystallization Characterizations

The obtained membrane flux is one of the most critical characterizations in the MD process [24,25]. It can be calculated using Equation (1), where *V*∗*ρ* is permeated mass, *A* is the active membrane area, and *t* is the operating time. In this study, a 100 mL measuring cylinder was used to measure the volume of permeate. Rejection rate is also an essential characterization of operation stability in the MD process [26,27,28]. The rejection rate was indicated by permeate conductivity in this study, which was measured using a conductivity meter (S470 Seven Excellence, Mettler Toledo, Switzerland). The lower the permeate conductivity, the higher the reject rate.

(1) J=(v∗ρ)/(A∗t)

The crystallization product is characterized by a recovery rate and grade, which can be calculated using Equations (2) and (3), respectively, where *m* is the weight of the target crystal product, *M* is the total weight of the target product, and *p* is the total weight of crystal product.

(2) η = mM∗100%

(3) γ=(m/p)∗100%

The crystals obtained from the single system were observed by stereomicroscope (XZT-CT, Shanghai Optics, Shanghai, China). Obtained mix crystals and membranes were determined using a field emission scanning electron microscope (FE-SEM, SUPRA55, Carl Zeiss AG, Oberkochen, Germany) and an energy-dispersive X-ray spectroscopy (EDS, Oxford INCA x-act, High Wycombe, UK). 

## 3. Results and Discussion

### 3.1. Single System Crystallization by MD Integrated with Crystallization 

**Figure 2:**
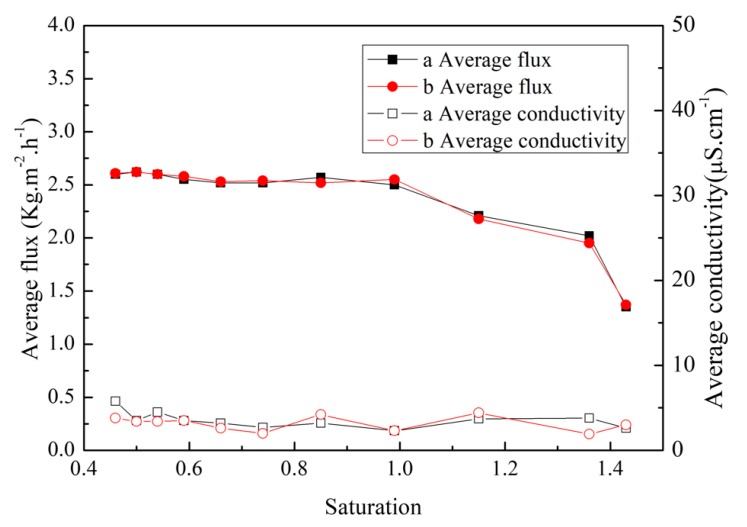
Evolution of flux and water yield conductivity during the membrane distillation (MD) operation. (a) ZnSO_4_ solution; (b) NiSO_4_ solution.

To investigate the feasibility of the MD integrated with crystallization process and to compare the effect of zinc ions with nickel ions, 20 wt % ZnSO_4_ solution and 20 wt % NiSO_4_ solution were used separately. The correspondences of saturation with mass score of ZnSO_4_ and NiSO_4_ at 65 °C are shown in Supplementary Tables S1 and S2. The variations in membrane flux and water yield conductivity as functions of saturation are represented in Figure 2. As can be seen, when the saturation was lower than 1 (42.64 wt % for ZnSO_4_ and 36.64 wt % for NiSO_4_), the fluxes of both solutions kept at 2.61 kg·m^−2^·h^−1^. When the saturation was higher than 1 and lower than 1.38 (50.63 wt % for ZnSO_4_ and 44.39 wt % for NiSO_4_), the fluxes showed a slow decline. Finally, when the saturation was higher than 1.38, the fluxes decreased sharply. During the whole MD operation, the water yield conductivity of both solutions remained below 10 μs·cm^−1^. Moreover, the rejection rates of heavy metal salt reached 99.99%.

Besides, the experiments involving ZnSO_4_ and NiSO_4_ solutions in air gap membrane distillation were consistent. Highly concentrated zinc and nickel ions did not have a different addition effect on the performance of MD, except that membrane fluxes were slightly lower than that of diluted solutions. Therefore, for the zinc and nickel mixed solution, it is possible to selectively separate the valuable metals according to their solubility differences at different temperatures. 

**Figure 3:**
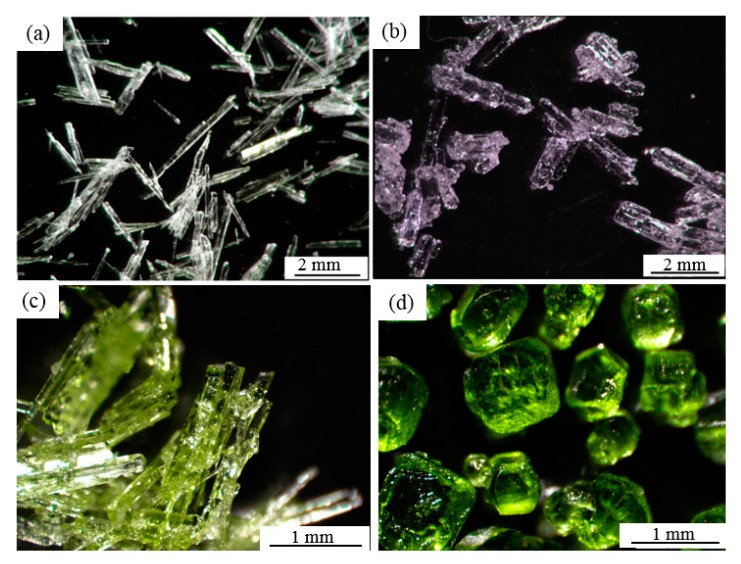
Morphology of crystalline products and commercial products. (**a**) ZnSO_4_·7H_2_O crystals by MD integrated with crystallization; (**b**) commercial ZnSO_4_·7H_2_O; (**c**) NiSO_4_·7H_2_O crystals by MD integrated with crystallization; (**d**) commercial NiSO_4_·6H_2_O.

After cooling crystallization at 10 °C, both the zinc and nickel sulfate crystalline products were obtained. According to crystallization chemistry, when the cooling temperature of the zinc sulfate solution is below 39 °C, and for the nickel sulfate solution, below 31.5 °C, the crystalline products will be ZnSO_4_·7H_2_O [29] and NiSO_4_·7H_2_O [30], respectively. In the view of the existence of carbonate and hydroxide ions (from the air and water) in the solution, the possible formation of carbonate and hydroxide precipitations is discussed in the supplemental information. The results indicate that it is impossible to form carbonate and hydroxide precipitations under the condition of the MD integrated with crystallization experiments. Obtained solid products were observed by stereomicroscope and compared with commercial products, respectively, as shown in Figure 3. The crystal products of zinc sulfate obtained from the MD integrated with crystallization process were colorless and needle-like, belonging to the orthorhombic system. The average size was 4 mm. Furthermore, its shape was more regular than the observed commercial products. On the other hand, the obtained nickel sulfate crystals were green, transparent, and belonged to the orthorhombic system. Their average size was 2 mm, and shapes were quite different from commercial NiSO_4_·6H_2_O, which was used to prepare the initial solution. 

### 3.2. Preferential Crystallization of Zinc Sulfate by MD Integrated with Crystallization

**Figure 4:**
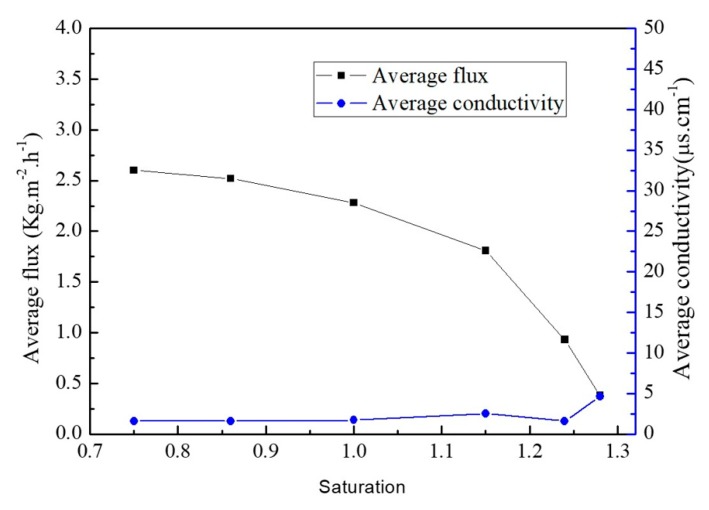
Evolution of flux and water yield conductivity during preferential crystallization of ZnSO_4._

In this experiment, 21.43 wt % ZnSO_4_ and 7.14 wt % NiSO_4_ mixed solution was used. MD integrated with crystallization experiments were performed to separate and obtain zinc sulfate crystal products selectively. As can be seen from Figure 4, membrane flux has an apparent decreasing trend with the increase of saturation. Additionally, when the saturation was higher than 1.15, membrane flux decreased rapidly. At the end of the process, when the saturation was 1.28, the flux was 0.38 kg·m^−2^·h^−1^. The membrane flux drop was probably caused by the combined action of solution vapor pressure drop, concentration polarization, temperature polarization, and membrane fouling [31,32,33]. During the whole MD process, the conductivity of permeate remained below 10 μs·cm^−1^. The rejection rate of heavy metal salt reached 99.99%. 

**Figure 5:**
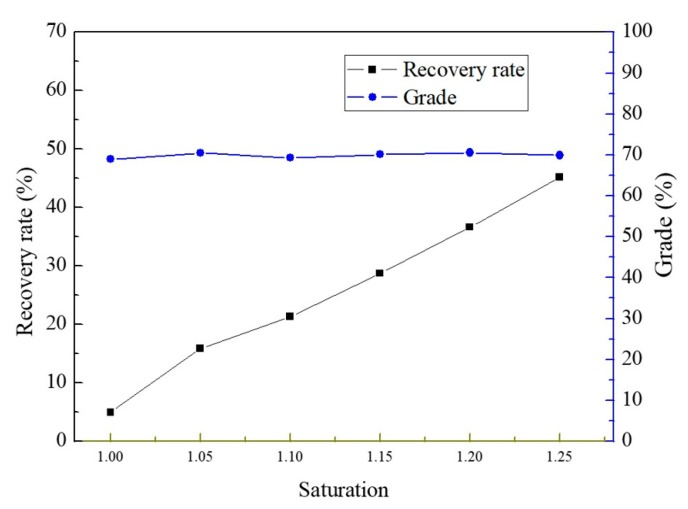
Recovery rate and grade of crystals produced by MD integrated with crystallization process at different saturation values.

After cooling crystallization at 10 °C, a mixture of zinc sulfate and nickel sulfate crystalline products were obtained. Figure 5 shows that the recovery rate and grade of ZnSO_4_·7H_2_O crystals varied at different saturation values during the MD integrated with crystallization process. As can be seen, with the increase of saturation, the recovery rate increased linearly. When the supersaturation was 1.18, the membrane flux was 1.5 kg·m^−2^·h^−1^, the recovery rate of zinc sulfate reached 35%. Nevertheless, the grade of crystals was about 70% and changed little at different saturations. 

**Figure 6:**
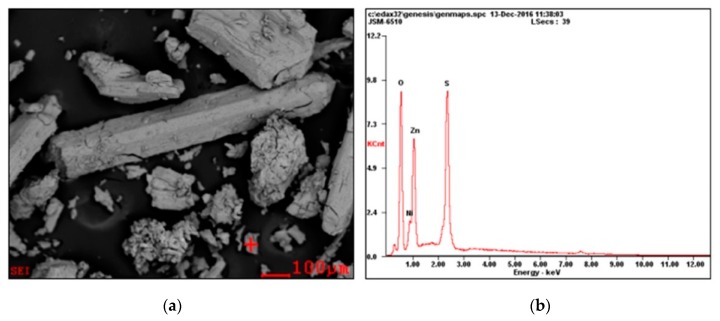
Scanning electron microscope (SEM) and energy-dispersive X-ray spectroscopy (EDS) diagrams of ZnSO_4_ crystal products. (**a**) SEM; (**b**) EDS.

After drying, the obtained crystal products were observed under SEM and measured by EDS. The results are shown in Figure 6. The composition of the main elements of crystals was 29.52% O, 33.80% S, 29.44% Zn, and 7.23% Ni. Both the results obtained from ICP-OES and EDS analysis suggest that this process does not have a zinc sulfate preferential crystallization due to the common-ion effect of SO_4_^2−^.

### 3.3. Preferential Crystallization of Nickel Sulfate by MD Integrated with Crystallization 

**Figure 7:**
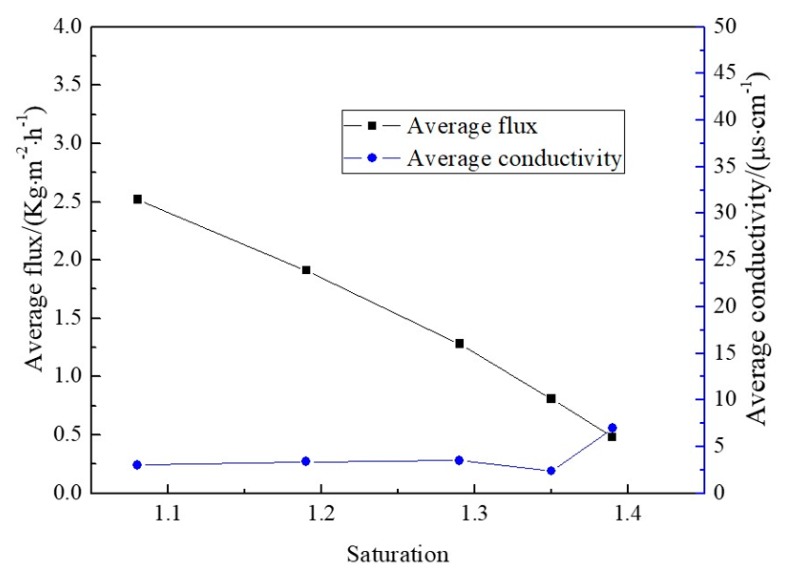
Evolution of flux and water yield conductivity during the preferential crystallization of NiSO_4_ experiment.

**Figure 8:**
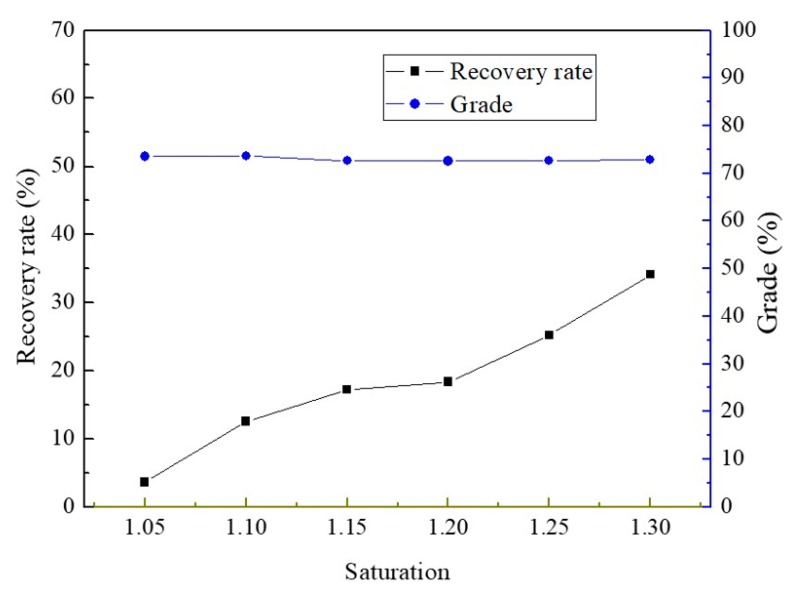
Recovery rate and grade of crystals produced by the MD integrated with crystallization process at different saturations.

The mass fractions of 10.34 wt % ZnSO_4_ and 20.69 wt % NiSO_4_ mixed solution were used to selectively separate and obtain nickel sulfate crystal products. As can be seen from Figure 7 and Figure 8, the preferential separation of NiSO_4_ showed the same tendency as the preferred separation of ZnSO_4_. The conductivity of permeate remained below 10 μs·cm^−1^ during the MD process, but the membrane flux showed a decline when the saturation increased. The recovery rate rose linearly with the increase of saturation. When the saturation was 1.18, the membrane flux was 1.5 kg·m^−2^·h^−1^, and the recovery rate was 35% by primary MD integrated with crystallization operating. However, the grade of crystals achieved 72% and changed little at different saturations.

**Figure 9:**
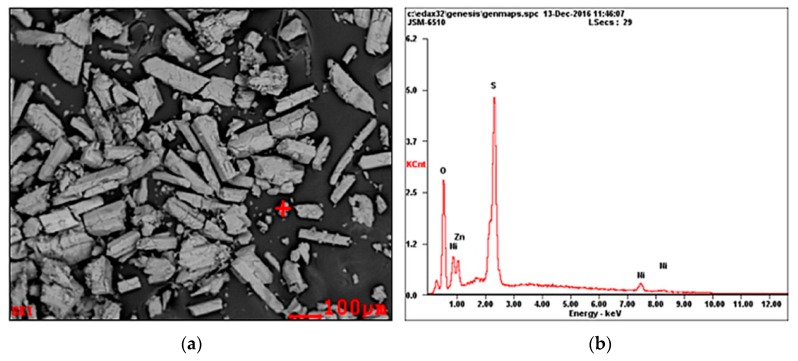
SEM and EDS diagrams of NiSO_4_ crystal products. (**a**) SEM; (**b**) EDS.

The obtained crystal products were also observed under SEM and measured by EDS. The results are shown in Figure 9. The main element composition of crystals was 12.57% O, 26.07% S, 6.52% Zn, and 54.85% Ni. The results showed that preferential crystallization of NiSO_4_ by MD integrated with crystallization was feasible while there was still 7.23% Ni in the ZnSO_4_·7H_2_O and NiSO_4_·7H_2_O salt products. 

### 3.4. Co-Crystallization of Zinc and Nickel Sulfate by MD Integrated with Crystallization

**Figure 10:**
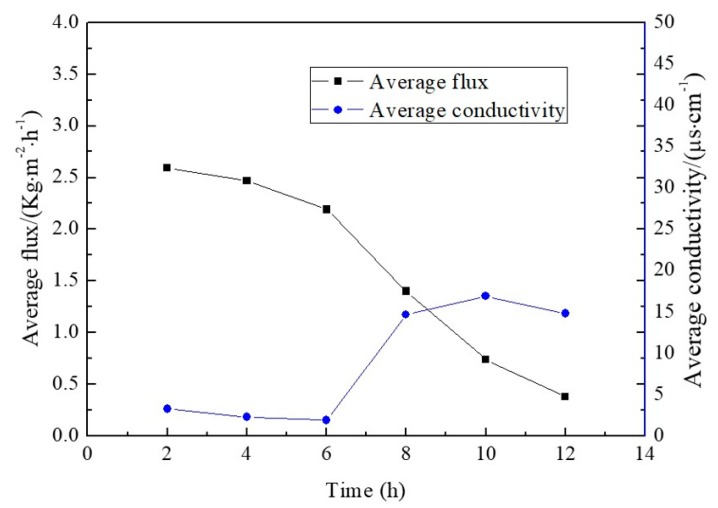
Evolution of membrane distillation flux and water yield conductivity of the mixed solution.

In these experiments, a ZnSO_4_ and NiSO_4_ mixed solution was used to investigate the co-crystallization. The mass fractions of ZnSO_4_ and NiSO_4_ were 20.00 wt % and 13.33 wt %, respectively. The variations in membrane flux and water yield conductivity as functions of filtration time are illustrated in Figure 10.

It can be seen from the figure, as the experiment goes on, the membrane flux reduces continuously. Six hours later, the membrane flux decreased rapidly, and the conductivity of permeate increased. Furthermore, after 12 h of operation, the flux was 0.38 kg·m^−2^·h^−1^. However, during the whole MD process, the conductivity of permeate remained below 20 μs·cm^−1^. The rejection rate of heavy metal salt still reached 99.99%. After natural drying, the crystals were dissolved in distilled water and then analyzed utilizing an ICP-OES. Among the products, the mass fraction of ZnSO_4_·7H_2_O was 64.96%, and the mass fraction of NiSO_4_·7H_2_O was 35.04%. 

### 3.5. Membrane Fouling

**Figure 11:**
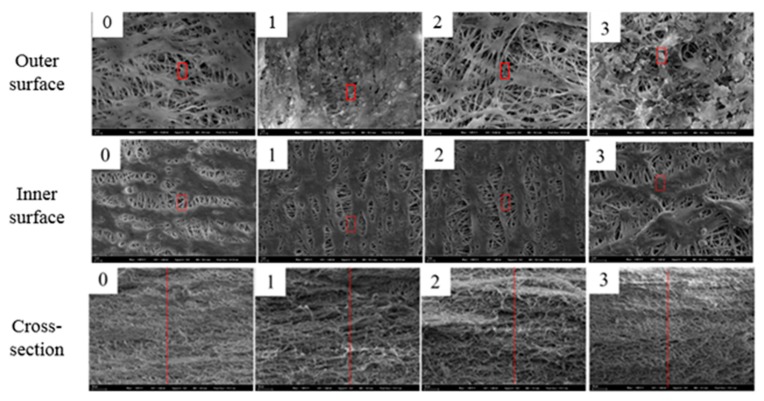
Morphologies of the membrane outer surface, inner surface, and cross-section. (**0**) new membrane; (**1**) zinc sulfate system; (**2**) nickel sulfate system; (**3**) mixed system.

As mentioned before, during the MD integrated with crystallization process, a decrease in the membrane flux was observed. After washing the system with tap and MILIQ water, membrane flux returned to its initial value and permeated conductivity again remained below 10 μs·cm^−1^. However, as an essential indicator of a comprehensive and in-depth evaluation of the membrane process [34,35,36], membrane surface morphology and surface deposits were investigated in this study. According to the differences in the treated liquids, tube membranes were labeled, as shown in Table 4. The working time of each membrane was calculated. The outer surface, inner surface, and cross-section of the membrane were observed by SEM, and the composition of the potential sediments was analyzed by EDS. In particular, to investigate how deeply the salts penetrate the pores of the membranes, EDS liner analysis was used instead of point analysis. In order to get neat cross-section samples, membranes were pre-treated with liquid nitrogen.As can be seen from Figure 11, the membranes used in this study have a density network structure. A small number of deposits can be seen on the outer surfaces of membrane 1 and membrane 3. This is mainly because of the highly concentrated solutions directly contacted with the outer surfaces. However, the network structure of the membrane surfaces can still be seen clearly. The deposition phenomenon on the membrane surfaces was not significant. Furthermore, there were no deposits found on the outer surfaces of membranes 0 and 1, nor the inner surfaces or cross-sections of all the membranes. The results of EDS liner analysis indicated that the deposits existed only on the membrane outer surfaces and did not penetrate deep into the membranes. Element and content analysis of the deposits on the outer membrane surface is shown in Table 5. The chemical formula of the PTFE membrane is (C_2_F_4_)_n_. Apart from the C and F elements, the amounts of sediment elements such as Zn, Ni, S, and O were relatively small on the surface of the membrane. All this proved that the membrane has excellent resistance to the fouling of highly concentrated zinc and nickel solutions.

## 4. Conclusions

The MD integrated with crystallization process has been confirmed to permit pure water and crystal products to be obtained from highly concentrated zinc and nickel mixed solutions. For the single element system, membrane fluxes remained at 2.61 kg·m^−2^·h^−1^ and showed a sharp decline until the saturation increased to 1.38. Water yield conductivity was maintained below 10 μs·cm^−1^. Highly concentrated zinc and nickel ions did not have extra influence on the membrane. For the mixed solutions, membrane flux showed a sharp decrease as an increase in saturation was observed, while the conductivity of permeate remained under 10 μs·cm^−1^ during the whole process. In this study, co-crystallization proves to be a better method due to the existence of the SO_4_^2−^ common-ion effect. Furthermore, membrane fouling studies suggest that the membrane has excellent resistance to the fouling of highly concentrated solutions.

The MD integrated with crystallization process has been demonstrated to be a promising technology in treating highly concentrated heavy metal solutions. However, nowadays, this technology is not used in the industry. Currently, one of the barriers to the implementation of MD integrated with crystallization is the price of the commercial membranes. It can be predicted that with the progress of membrane technology and materials science, MD integrated with crystallization technology will be widely used in the industry.

## Figures and Tables

**Table 1:** Solubility and corresponding mass fraction of ZnSO_4_ and NiSO_4_ at different temperatures [22].

Material	Temperature/°C	Solubility	Mass Fraction/%
ZnSO_4_	10	47.20	32.07
	65	74.33	42.63
NiSO_4_	10	32.00	24.24
	65	57.83	36.64

**Table 2:** Three different methods to treat mixed solutions.

Ratios of Zinc Sulfate and Nickle Sulfate	Method
3:1	Preferential crystallization of Zinc sulfate
0.5:1	Preferential crystallization of Nickle sulfate
1.5:1	Mixed co-crystallization

**Table 3:** Parameters of membrane material and module.

Membrane and Module	Properties
Diameter/mm	22
Membrane thickness/mm	0.300
Nominal pore size/µm	0.083
Maximum pore size/µm	0.211
Contact angle/deg	115.7
Tensile stress at break/MPa	54
Strain at break/%	240
Effective length/m	0.308
Effective membrane area/(m^2^)	0.213

**Table 4:** Information of the studied membranes.

Number	0	1	2	3
Composition of the treated solutions	New membrane	Zinc sulfate	Nickel sulfate	Zinc and nickel sulfate
Work time	0 h	515 h	22 h	95 h

**Table 5:** Elements and contents of deposits on the membrane outer surface.

Membrane Number/Element Content %	C	O	F	S	Zn	Ni
0	23.33	-	76.67	-	-	0
1	24.39	-	73.63	0.32	3.86	-
2	19.99	-	80.01	-	-	-
3	24.99	6.15	56.58	-	11.2	1.08

## Supplementary Materials

The following are available online at https://www.mdpi.com/2077-0375/10/1/19/s1, Table S1: Correspondence of saturation with the mass fraction of ZnSO_4_ solution at 65 °C. Table S2: Correspondence of saturation with the mass fraction of NiSO_4_ solution at 65 °C.
