# Supplementary material for: Separation and Recycling of Concentrated Heavy Metal Wastewater by Tube Membrane Distillation Integrated with Crystallization
Source: Membranes (Basel). 2020 Jan 20;10(1):19. doi: 10.3390/membranes10010019 (PMC7022982; doi:10.3390/membranes10010019)
Supplement: Supplementary file 1 [file membranes-10-00019-s001.pdf]

*membranes*

# Supplementary Materials: Separation and Recycling of Concentrated Heavy Metal Wastewater by Tube Membrane Distillation Integrated with Crystallization

**Xiang-Yang Lou [1,2,3], Zheng Xu [1,4], An-Ping Bai [5], Montserrat Resina-Gallego [2] and Zhong-Guang Ji [1,4,*]**

1 National Engineering Lab. of Biohydrometallurgy, GRINM Technology Group Co., Ltd., Beijing 101407, China; Xiangyang.Lou@uab.cat (X.-Y.L.); xzh63@126.com (Z.X.)
2 GTS Research Group, Department of Chemistry, Faculty of Science, Universitat Autònoma de Barcelona, Bellaterra 08290, Spain; montserrat.resina@uab.cat
3 General Research Institute for Nonferrous Metals, Beijing 100088, China
4 GRINM Resources and Environmental Tech. Co., Ltd., Beijing 101407, China
5 Beijing Vocational College of Labor and Social Security, Beijing 102200, China; baianping1992@163.com
* Correspondence: jizhongguang@grinm.com; Tel.: +86-010-8224-1783

**Table 1.** Correspondence of saturation with the mass fraction of $ZnSO_4$ solution at 65 °C.

| Saturation | Mass Fraction/% |
|---|---|
| 0.34 | 20 |
| 1.0 | 42.64 |
| 1.1 | 44.98 |
| 1.2 | 47.14 |
| 1.3 | 49.14 |
| 1.38 | 50.63 |
| 1.4 | 51.00 |
| 1.5 | 52.72 |
| 1.6 | 54.32 |
| 1.7 | 55.82 |
| 1.8 | 57.23 |
| 1.9 | 58.55 |

**Table 2.** Correspondence of saturation with the mass fraction of $NiSO_4$ solution at 65 °C.

| Saturation | Mass Fraction/% |
|---|---|
| 0.43 | 20 |
| 1.0 | 36.64 |
| 1.1 | 38.88 |
| 1.2 | 40.97 |
| 1.3 | 42.92 |
| 1.38 | 44.39 |
| 1.4 | 44.74 |
| 1.5 | 46.45 |
| 1.6 | 48.06 |
| 1.7 | 49.57 |
| 1.8 | 51.00 |
| 1.9 | 52.35 |

## 1. Possibility Discussions of Carbonate and Hydroxide Salts during the Experiments

For hydroxide: pK [$Zn(OH)_2$] = $3.3 \times 10^{-13}$; pK [$Ni(OH)_2$] = $3.0 \times 10^{-16}$. In our experiments, the pH values are 4–5. So, the concentration of $H^+$ is higher than $10^{-5}$ M and lower than $10^{-4}$ M. The concentration of $OH^-$ is lower than $10^{-9}$ M.

In order to form the crystal of $Zn(OH)_2$, the concentration of $Zn^{2+}$ should be higher than $3.3 \times 10^5$ M ($2.16 \times 10^7$ g·L$^{-1}$). For the crystal of $Ni(OH)_2$, the concentration of $Ni^{2+}$ should be higher than $3.0 \times 10^2$ M ($1.76 \times 10^4$ g·L$^{-1}$). So, it is impossible to form the hydroxide precipitations in the experiments.

For carbonate: pK [$ZnCO_3$] = $1.7 \times 10^{-11}$; pK [$NiCO_3$] = $1.3 \times 10^{-7}$.

$$CO_2\,(aq) \rightleftharpoons H_2CO_3\,(aq) \quad K_1 = 1.70 \times 10^{-3} \tag{S1}$$

$$HCO_3^-\,(aq) \rightleftharpoons H^+\,(aq) + CO_3^{2-}\,(aq) \quad K_3 = 5.01 \times 10^{-11} \tag{S2}$$

So,

$$CO_2\,(aq) \rightleftharpoons 2H^+\,(aq) + CO_3^{2-}\,(aq) \quad K = K_1 \times K_2 \times K_3 = 2.13\times10^{-17} \tag{S3}$$

The current, average concentration of $CO_2$ is 387 ppm, thus $1.32 \times 10^{-5}$ M; the concentration of $H^+$ is above $10^{-5}$ M. So, the concentration of $CO_3^{2-}$ is below $1.61 \times 10^{-15}$ M.

In order to form the crystal of $ZnCO_3$, the concentration of $Zn^{2+}$ should be higher than $1.06 \times 10^3$ M ($6.93 \times 10^4$ g·L$^{-1}$). For the crystal of $NiCO_3$, the concentration of $Ni^{2+}$ should be higher than $8.07 \times 10^7$ M ($4.74 \times 10^9$ g·L$^{-1}$).

So, it is impossible to form carbonate precipitations in the experiments.

**Reference:**


1. Fengcheng, S. *Basic environmental engineering chemical experiment, 2nd ed.*; Wunan Publishing: Taiwan, China, 2017; pp. 91–93.
2. Carbon Dioxide as an Acid. Available online: http://butane.chem.uiuc.edu/pshapley/GenChem1/L25/1.html (accessed on 13 01 2020).


© 2020 by the authors. Submitted for possible open access publication under the terms and conditions of the Creative Commons Attribution (CC BY) license (http://creativecommons.org/licenses/by/4.0/).
